# Annotations

**Published:** 1890-08-16

**Authors:** 


### Annotations.

**Execution by Electricity.**

The execution of Kemmler at New York last week by electricity does not seem to have been either so speedy or so painless as persons of humane sentiments desired. It is a pity the first experiment of putting a man to death by that means was tried in America. The American public are either greatly misrepresented or excessively childish and inconsequent. Even if Kemmler actually suffered a little more than was anticipated—though there is no reason to suppose he really suffered at all—yet every writer of the sensational newspaper paragraphs which have appeared, and every reader of them, must have known that the object of trying the method of execution by electricity was an altogether humane one; and if the first experiment was a partial failure what need was there for the senseless howls and artificial rage which greeted all who took any active part in the business. Criminals must be executed, and if a more humane method than that of hanging or beheading can be devised, there are the best reasons for trying it, even though there is always the possibility that the first person experimented upon may have a rather unhappy experience. Whatever else has not emerged as the result of the trial of the new method, some exceedingly childish and discreditable behaviour on the part of a section of the American public has been very conspicuous. Many people, influenced by the clamour, will be for going back to hanging or beheading at once. That, however, would certainly not be a rational proceeding. It is desirable, at least, to try one or more experiments. There ought to be no difficulty in avoiding the mistakes which marred the effectiveness of the Kemmler execution. But even though it should ultimately be demonstrated that execution by electricity is undesirable there are still other methods than those of hanging and beheading which may be tried. Our own fellow-countryman, Dr. Benjamin Ward Richardson, has devised a lethal chamber whereby animals are put to death quietly, easily, and without consciousness of impending doom. Why should some such method not be used for criminals? But why, indeed, should every criminal be executed in one and the same stereotyped way? The resources of civilization are surely equal to the invention of half a dozen different methods! And the criminal himself might, without any harm to society, be allowed to choose the manner of his death. If the execution of Kemmler answers no other good purpose, it at least calls attention to the fact that our present modes of execution are rude and coarse, and may with propriety be now reconsidered. Death itself is a sufficient penalty for the very worst of crimes, and there is every reason for making it as painless, speedy, and as little revolting as possible.

**"A Reprehensible Practice."**

The daily press has acquainted all the world with a practice which it is stated, has long been common among a certain class of doctors. A Midland practitioner was, it appears, absent from home on his holiday. He had left his unqualified assistant behind him in charge of his practice. One patient was so very ill that the doctor did not expect him to survive many days, and therefore he left on his departure a signed death certificate, which the unqualified assistant filled up when the patient's death actually occurred. There appears to have been no real harm done. The certificate bore the actual cause of death; and there is no reason for believing that the doctor who signed it had not himself attended the case and made the diagnosis. But every death certificate bears on its face the statement that the doctor signing it has actually seen the dead man either on the day of his death or on some other stated day near it. In this case it was certified that the doctor had seen the patient on a certain day, whereas the fact was that on that very day the doctor was absent from home and could not possibly have seen him. Indeed, when the case was brought before the magistrate there was no pretence made that the doctor had himself seen the case, but only that the unqualified assistant had. Here, then, in plain terms, is an instance of a duly qualified medical practitioner putting his name to a lie; and of his unqualified assistant availing himself of his master's falsehood; and both, apparently, without any scruple whatever. The Registrar said that this was an example of a common practice. Now, medical men would object very much to being called illiterate, or to being classed with persons who are not gentlemen. Is it an educated man's or a gentleman's action to lie deliberately; to go so far as to put his name to a lie that is going to be told some days, or possibly weeks hence? The morals of the horsefair, we believe, admit of lying. But we have yet to learn that lying is a part of the gentleman's social or business code. For a servant to lie to her mistress is considered disgraceful and shocking. The duly qualified medical man of whom we write seemed to see nothing disgraceful in his performance. It suited his convenience to leave his unqualified assistant in his place during his absence; and, if the arrangement necessitated the telling of a lie or two, that was nothing at all. It is difficult to say anything which will reach doctors of so uncultured and undignified a class as this. Probably the last consideration which would be likely to have any weight with them would be a moral one. A lie to them is only base and vulgar when it is found out. That anyone should object to it on the score of its immorality would probably be a source of amusement to them. If self-respect and a regard for honourable conduct be not sufficient to restrain such doctors from such deeds, we can only hope that the law will step in with an adequate penalty. Persons who are not readily touched when personal character only is at stake are often exceedingly sensitive when the pocket or the skin is the object of attack.

**The Wrongs of Private Nurses.**

A "Hospital Life Governor" wrote to us some time ago on the subject of nurses' wrongs. He wished us to publish his letter, and we should have been glad to do so if he had complied with the conditions which are standing orders in all newspaper offices and given us his name. We had no to the writer and to all impartial persons that newspapers desire to make the name public; but it must be quite evident cannot publish letters unless they know who the writers are, and that for very obvious reasons. How, for instance, is the editor to know whether the writer of the particular letter in question is really a life governor or not? And supposing the letter had been published, and a critical reader had denied that it was written by a life governor, and asserted that the real writer was a member of the hospital staff: how could the editor have proved it to be otherwise? The fact seems to be this, that the "Hospital Life Governor" is anxious to put upon the editor's shoulders a responsibility which he will not bear himself. He says that nurses suffer grievous wrongs at private nursing institutions; he asks us to expose those institutions, and because we have not published his letter he charges us with "systematic concealment of the wrongs of the nurses," and with being "quite content that those wrongs should be perpetuated." Now we have never concealed the wrongs of nurses either systematically or otherwise; and we shall not be the least content so long as we know of a single nurse who suffers from unrighted wrongs. The boot, indeed, appears to be on the other leg. A "Hospital Life Governor" knows, or thinks he knows, of some nurses who are badly treated at certain private nursing institutions. If he is sure of his facts is it not his plain duty to tell the public of those facts; to give the names of the institutions, of the irresponsible matrons, and of the illtreated nurses; and also to give the dates and circumstances of the injurious treatment? We offer him the columns of The Hospital in which to set forth his case; and if he does not choose to accept the offer, with its attendant responsibility, we cannot but accuse him of "systematic concealment of the wrongs of the nurses, who are at the mercy of irresponsible matrons and sisters," and affirm that "it is very certain that he is quite content that those wrongs should be perpetuated." What kind of champion of meritorious and injured women is he who will only consent to try to redress their grievances by means of a third party, carefully refraining from taking the least shadow of responsibility upon his own shoulders? If his charges are well founded, we are willing that "A Hospital Life Governor" should play Sancho Panza to our Don Quixote, but only on condition that we first know who it is we have to deal with.

